# (4*R*,5*R*,6*S*,7*R*,8*S*,9*R*,10*S*,13*S*)-7,8β-Epoxy­momilactone-A

**DOI:** 10.1107/S1600536808010556

**Published:** 2008-04-23

**Authors:** Rana Shabnam Habib, Muhammad Jamshaid, M. Nawaz Tahir, Tahir Javed Khan, Islam Ullah Khan

**Affiliations:** a University College of Pharmacy, University of the Punjab, Lahore 54590, Pakistan; b University of Sargodha, Department of Physics, Sargodha, Pakistan; c Government College University, Department of Chemistry, Lahore, Pakistan

## Abstract

The title compound, C_20_H_26_O_4_, was extracted from *Leucas Urticifolia*, a wild Lamiaceae herb distributed in the Punjab, Baluchistan, Sindh and the Rajputana desert of Pakistan. The plant is utilized for various medicinal applications by the local community. The title compound is based on the pimarane–diterpene skeleton. The mol­ecule exhibits an ep­oxy ring fused to momilactone-A, leading to a penta­cyclic mol­ecular structure. The absolute configuration was assigned by comparison with the crystal structure of momilactone, but needs further verification. The crystal structure is governed by four inter­molecular hydrogen-bond inter­actions of the C—H⋯O type.

## Related literature

For related literature, see: Bhattecharjee (2004 ▶); Germain & Deslongchamps (2002 ▶); Kato *et al.* (1973 ▶); Kiritikhar & Basu (2005 ▶); Misra *et al.* (1992 ▶, 1993 ▶, 1995 ▶).
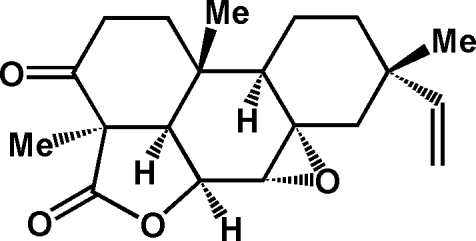


## Experimental

### 

#### Crystal data

C_20_H_26_O_4_
*M* *_r_* = 330.41
Orthorhombic, 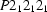

*a* = 6.3996 (7) Å
*b* = 13.1759 (3) Å
*c* = 20.854 (1) Å
*V* = 1758.4 (2) Å^3^
*Z* = 4
Mo *K*α radiation
μ = 0.09 mm^−1^
*T* = 296 (2) K
0.25 × 0.10 × 0.09 mm

#### Data collection

Bruker Kappa APEXII CCD diffractometer
Absorption correction: multi-scan (*SADABS*; Bruker, 2005 ▶) *T*_min_ = 0.980, *T*_max_ = 0.990
19288 measured reflections
2635 independent reflections
1202 reflections with *I* > 2σ(*I*)
*R*_int_ = 0.075

#### Refinement

*R*[*F* ^2^ > 2σ(*F* ^2^)] = 0.050
*wR*(*F* ^2^) = 0.117
*S* = 1.03
2635 reflections
223 parameters
H atoms treated by a mixture of independent and constrained refinement
Δρ_max_ = 0.14 e Å^−3^
Δρ_min_ = −0.17 e Å^−3^

### 

Data collection: *APEX2* (Bruker, 2007 ▶); cell refinement: *APEX2*; data reduction: *SAINT* (Bruker, 2007 ▶); program(s) used to solve structure: *SHELXS97* (Sheldrick, 2008 ▶); program(s) used to refine structure: *SHELXL97* (Sheldrick, 2008 ▶); molecular graphics: *ORTEP-3 for Windows* (Farrugia, 1997 ▶) and *PLATON* (Spek, 2003 ▶); software used to prepare material for publication: *WinGX* (Farrugia, 1999 ▶) and *PLATON*.

## Supplementary Material

Crystal structure: contains datablocks global, I. DOI: 10.1107/S1600536808010556/im2057sup1.cif

Structure factors: contains datablocks I. DOI: 10.1107/S1600536808010556/im2057Isup2.hkl

Additional supplementary materials:  crystallographic information; 3D view; checkCIF report

## Figures and Tables

**Table 1:** Hydrogen-bond geometry (Å, °)

*D*—H⋯*A*	*D*—H	H⋯*A*	*D*⋯*A*	*D*—H⋯*A*
C2—H2*B*⋯O2^i^	0.97	2.58	3.356 (5)	137
C7—H7⋯O4^ii^	0.98	2.58	3.164 (4)	118
C11—H11*A*⋯O1^iii^	0.97	2.55	3.420 (5)	149
C14—H14*B*⋯O1^iv^	0.97	2.52	3.486 (5)	178

Symmetry codes: (i) 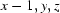; (ii) 
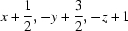
; (iii) 
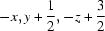
; (iv) 
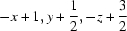
.

**Fig. 1.**
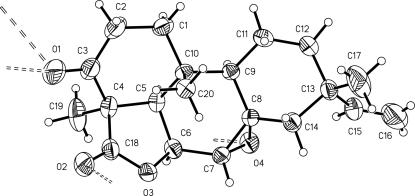
ORTEP diagram of the title compound (I) with displacement ellipsoids drawn at 30% probability level; H-atoms are shown by small circles of arbitrary radii; dashed lines indicate the direction of intermolecular C—H···O hydrogen bonding.

## supplementary crystallographic information

### Comment

*Leucas urticaefolia* is a wild Lamiaceae herb distributed in the Punjab, Baluchistan, Sindh and Rajputana desert of Pakistan. At Gomawal in Baluchistan, the plant is used as a cure for fever. It's local name in Gujerati is Kubo (Kiritikhar & Basu, 2005) and in Tilla Gogian of the Potohar region, it is known as Goma or Guldora. The decoction of the leaves and apical shoots with gur is used locally as an abortifacient up to 3 months of pregnancy. Infusion of the flowers are used in skin diseases. It is also used to treat piles. Other plants of the genera are also used in local remedies, e.g, the flowers of *Leucas cephalotes* are used to treat cold and cough while the entire plant has stimulant and insecticidal properties (Bhattecharjee, S.K., 2004). No work has been recorded for *Leucas Urticaefolia* although other plants of the genera have shown biologically and physiologically interesting classes of compounds (Misra *et al.*, 1992; Misra *et al.*, 1993; Misra *et al.*, 1995).

Momilactone was originally extracted from the seed husk (Kato *et al.*, 1973). The crystal structure was determined and the assignment of the absolute configuration was performed by several spectroscopic techniques. Synthesis of racemic (±)-Momilactone A and a related structure has been reported (Germain & Deslongchamps, 2002). The title compound (I), C_20_H_26_O_4_, is related to Momilactone A differing by one additional epoxy ring system therefore resulting in a pentacyclic molecular skeleton. There are three six membered rings: A (C1 to C5, C10), B (C5 to C10) and C (C8,C9,C11 to C14). One five membered ring D (C4 to C6, O3, C18) with the lactone functional group is fused to ring A and ring B. The three membered epoxy ring E (C7, C8, O4) is fused to ring B in β-position. In analogy to the molecular structure of Momilactone the title compound was refined in the same absolute configuration with the chiral centers in the molecule being C4(*R*), C5(*R*), C6(*S*), C7(*R*), C8(*S*), C9(*R*), C10(*S*) and C13(*S*). Nevertheless, this configuration of course cannot be determined reliably using the experimental conditions of this investigation. Due to the methyl group attached to the common vertex of ring A and B at C10, these rings show an envelope conformation with C10 in an almost identical distance of -0.789 (4) Å from both planes. The dihedral angle between the planes (C1 to C5) and (C5 to C9) is 35.1 (2)° while the epoxy ring (C7, C8, O4) encloses dihedral angles of 39.8 (2)° and 74.9 (2)° with them, respectively. The puckering parameters (Cremer & Pople, 1975) for ring A and B are Q = 0.570 (3) Å and 0.584 (3) Å, θ = 119.8 (4)° and 122.1 (3)° and φ = 122.6 (4)° and 113.8 (4)°, respectively. Ring C exhibits a twist conformation with the planes (C8, C9, C12, C14) and (C11, C12, C13, C14) showing a dihedral angle of 51.1 (2)°. For ring D also an envelope conformation is observed with C5 at a distance of -0.485 (5) Å from the plane (C4, C18, O3, C6). The puckering parameters (Cremer & Pople, 1975) for ring C are Q = 0.724 (4) Å, θ = 96.3 (3)° and φ = 315.3 (3)°.

In (I), the bond lengths C3=O1 and C18=O2 have identical values [1.198 (4), 1.197 (4) Å]. The C=C bond length [C15 = C16] is 1.281 (7) Å, while the C—C bond distance for methyl C-atoms from the ring carbons have nearly same value of 1.537 (4) Å. These values are similar to the reported submitted (CCDC No. 172789) by Germain & Deslongchamps, 2002. In the crystal structure, the asymmetric unit is linked to four neighboring molecules through intermolecular C—H···O hydrogen bonds (Table 1). These H-bonds (Fig. 1) seem to be effective in the stabilization of the structure. There is no significant π-π interaction.

### Experimental

*Leucas Urticaefolia* was collected from the hills of Khanaspur, Pakistan. The plants were separated into stems (1.03 kg), inflorescence (256.56 g), leaves (812.34 g) and roots (421.34 g). Each of these fractions was dried under shade, powdered and extracted sequentially at room temperature for 72 h in each of the solvents such as hexane, chloroform, ethanol and water. Solvents were removed under reduced pressure. Each extract was extracted successively with n-hexane, chloroform, methanol and water as eluent in increasing order of polarity. Aluminium sheets precoated with silica gel 60 F254 (0.2 mm thick, E Merck) were used for TLC. Column chromatography was carried out on silica gel, 70–230 mesh. The title compound was taken from the water extract of the leaves.

### Refinement

All H-atoms were found in Fourier synthesis and refined initially. However H atoms positioned geometrically resulted in the same values of refinement parameters.

In final refinement the coordinates of H-atoms connected to C16 were refined. The rest of H-atoms were positioned geometrically, with C-H = 0.93, 0.97, 0.96 Å for C15, methylene and methyl C-atoms and constrained to ride on their parent atoms. The thermal parameter of methyl H-atoms was taken 1.5 times while for all other H-atoms it was taken 1.2 times of the parent atoms.

### Crystal data

**Table:**

C_20_H_26_O_4_	*F*_000_ = 712
*M**_r_* = 330.41	*D*_x_ = 1.248 Mg m^−^^3^
Orthorhombic, *P*2_1_2_1_2_1_	Mo *K*α radiation λ = 0.71073 Å
Hall symbol: P 2ac 2ab	Cell parameters from 1295 reflections
*a* = 6.3996 (7) Å	θ = 1.6–28.6º
*b* = 13.1759 (3) Å	µ = 0.09 mm^−^^1^
*c* = 20.854 (1) Å	*T* = 296 (2) K
*V* = 1758.4 (2) Å^3^	Needle, colourless
*Z* = 4	0.25 × 0.10 × 0.09 mm

### Data collection

**Table:**

Bruker Kappa APEX2 CCD diffractometer	2635 independent reflections
Radiation source: fine-focus sealed tube	1202 reflections with *I* > 2σ(*I*)
Monochromator: graphite	*R*_int_ = 0.075
Detector resolution: 7.30 pixels mm^-1^	θ_max_ = 28.9º
*T* = 296(2) K	θ_min_ = 2.5º
ω scans	*h* = −8→8
Absorption correction: multi-scan(SADABS; Bruker, 2005)	*k* = −17→14
*T*_min_ = 0.980, *T*_max_ = 0.990	*l* = −28→28
19288 measured reflections	

### Refinement

**Table:**

Refinement on *F*^2^	Secondary atom site location: difference Fourier map
Least-squares matrix: full	Hydrogen site location: inferred from neighbouring sites
*R*[*F*^2^ > 2σ(*F*^2^)] = 0.050	H atoms treated by a mixture of independent and constrained refinement
*wR*(*F*^2^) = 0.117	*w* = 1/[σ^2^(*F*_o_^2^) + (0.0443*P*)^2^ + 0.0772*P*] where *P* = (*F*_o_^2^ + 2*F*_c_^2^)/3
*S* = 1.03	(Δ/σ)_max_ < 0.001
2635 reflections	Δρ_max_ = 0.14 e Å^−^^3^
223 parameters	Δρ_min_ = −0.17 e Å^−^^3^
Primary atom site location: structure-invariant direct methods	Extinction coefficient: ?

### Special details

**Table:**

Geometry. All e.s.d.'s (except the e.s.d. in the dihedral angle between two l.s. planes) are estimated using the full covariance matrix. The cell e.s.d.'s are taken into account individually in the estimation of e.s.d.'s in distances, angles and torsion angles; correlations between e.s.d.'s in cell parameters are only used when they are defined by crystal symmetry. An approximate (isotropic) treatment of cell e.s.d.'s is used for estimating e.s.d.'s involving l.s. planes.
Refinement. Refinement of *F*^2^ against ALL reflections. The weighted *R*-factor *wR* and goodness of fit *S* are based on *F*^2^, conventional *R*-factors *R* are based on *F*, with *F* set to zero for negative *F*^2^. The threshold expression of *F*^2^ > σ(*F*^2^) is used only for calculating *R*-factors(gt) *etc*. and is not relevant to the choice of reflections for refinement. *R*-factors based on *F*^2^ are statistically about twice as large as those based on *F*, and *R*- factors based on ALL data will be even larger. The low data parameter ratio is due to the small size of the crystal as well as to the absence of heavy atoms.

### Fractional atomic coordinates and isotropic or equivalent isotropic displacement parameters (Å^2^)

**Table:**

	*x*	*y*	*z*	*U*_iso_*/*U*_eq_	
O1	0.2901 (4)	0.5384 (2)	0.77739 (11)	0.1276 (11)	
O2	0.6901 (4)	0.54820 (17)	0.70678 (11)	0.0895 (7)	
O3	0.6511 (3)	0.64702 (13)	0.62196 (9)	0.0641 (5)	
O4	0.4101 (3)	0.82859 (12)	0.50957 (8)	0.0624 (5)	
C1	0.0673 (4)	0.7697 (2)	0.70647 (13)	0.0687 (8)	
H1A	−0.0511	0.7524	0.6796	0.082*	
H1B	0.0347	0.8324	0.7288	0.082*	
C2	0.1031 (5)	0.6849 (2)	0.75567 (14)	0.0848 (10)	
H2A	0.1551	0.7159	0.7947	0.102*	
H2B	−0.0316	0.6551	0.7657	0.102*	
C3	0.2466 (5)	0.6019 (3)	0.73828 (15)	0.0739 (9)	
C4	0.3374 (5)	0.5948 (2)	0.67075 (12)	0.0577 (7)	
C5	0.2923 (4)	0.68587 (18)	0.62796 (11)	0.0499 (7)	
H5	0.1673	0.6716	0.6024	0.060*	
C6	0.4805 (4)	0.68849 (19)	0.58381 (11)	0.0553 (7)	
H6	0.4545	0.6434	0.5473	0.066*	
C7	0.5436 (4)	0.7907 (2)	0.55901 (11)	0.0535 (7)	
H7	0.6937	0.8028	0.5537	0.064*	
C8	0.4140 (4)	0.87944 (18)	0.57182 (11)	0.0462 (6)	
C9	0.2192 (4)	0.86667 (18)	0.61280 (11)	0.0459 (6)	
H9	0.1100	0.8397	0.5846	0.055*	
C10	0.2590 (4)	0.78561 (18)	0.66466 (11)	0.0449 (6)	
C11	0.1424 (5)	0.9704 (2)	0.63641 (13)	0.0654 (8)	
H11A	0.0037	0.9639	0.6547	0.078*	
H11B	0.2354	0.9960	0.6694	0.078*	
C12	0.1373 (5)	1.0436 (2)	0.57995 (14)	0.0672 (8)	
H12A	0.0686	1.1058	0.5932	0.081*	
H12B	0.0551	1.0139	0.5457	0.081*	
C13	0.3552 (5)	1.06931 (19)	0.55425 (14)	0.0631 (8)	
C14	0.5096 (4)	0.98319 (19)	0.57133 (14)	0.0608 (8)	
H14A	0.6235	0.9839	0.5406	0.073*	
H14B	0.5684	0.9968	0.6133	0.073*	
C15	0.3421 (6)	1.0775 (3)	0.48310 (17)	0.0956 (12)	
H15	0.3070	1.0178	0.4619	0.115*	
C16	0.3716 (10)	1.1536 (5)	0.4474 (3)	0.171 (3)	
C17	0.4321 (6)	1.1672 (2)	0.5862 (2)	0.1304 (16)	
H17A	0.4403	1.1573	0.6317	0.196*	
H17B	0.3363	1.2213	0.5770	0.196*	
H17C	0.5679	1.1843	0.5699	0.196*	
C18	0.5739 (5)	0.5923 (2)	0.67153 (14)	0.0635 (8)	
C19	0.2609 (7)	0.4944 (2)	0.64183 (16)	0.1028 (13)	
H19A	0.1109	0.4939	0.6409	0.154*	
H19B	0.3099	0.4389	0.6675	0.154*	
H19C	0.3139	0.4876	0.5990	0.154*	
C20	0.4458 (4)	0.81615 (19)	0.70649 (11)	0.0535 (7)	
H20A	0.5665	0.8259	0.6799	0.080*	
H20B	0.4735	0.7635	0.7372	0.080*	
H20C	0.4143	0.8782	0.7286	0.080*	
H16A	0.378 (5)	1.143 (2)	0.4045 (13)	0.080*	
H16B	0.397 (5)	1.214 (2)	0.4727 (13)	0.080*	

### Atomic displacement parameters (Å^2^)

**Table:**

	*U*^11^	*U*^22^	*U*^33^	*U*^12^	*U*^13^	*U*^23^
O1	0.124 (2)	0.153 (2)	0.1054 (19)	0.0407 (19)	0.0296 (18)	0.0790 (18)
O2	0.0755 (17)	0.0982 (17)	0.0950 (15)	0.0171 (13)	−0.0114 (13)	0.0445 (13)
O3	0.0669 (13)	0.0666 (12)	0.0588 (11)	0.0172 (10)	0.0070 (11)	0.0134 (10)
O4	0.0828 (15)	0.0634 (12)	0.0409 (10)	0.0064 (10)	−0.0036 (9)	0.0013 (9)
C1	0.0527 (19)	0.082 (2)	0.0713 (19)	−0.0007 (16)	0.0122 (16)	0.0101 (18)
C2	0.072 (2)	0.105 (3)	0.078 (2)	−0.011 (2)	0.0138 (18)	0.028 (2)
C3	0.061 (2)	0.084 (2)	0.077 (2)	−0.0048 (19)	−0.0003 (18)	0.0255 (19)
C4	0.063 (2)	0.0541 (17)	0.0558 (18)	−0.0116 (16)	−0.0113 (16)	0.0105 (14)
C5	0.0542 (18)	0.0468 (16)	0.0487 (14)	−0.0096 (13)	−0.0153 (13)	0.0047 (13)
C6	0.074 (2)	0.0522 (18)	0.0399 (14)	0.0088 (15)	−0.0037 (15)	−0.0001 (13)
C7	0.0567 (18)	0.0583 (18)	0.0455 (15)	0.0058 (14)	0.0061 (14)	0.0049 (13)
C8	0.0454 (16)	0.0496 (16)	0.0435 (14)	0.0008 (13)	−0.0021 (13)	−0.0003 (12)
C9	0.0398 (16)	0.0517 (16)	0.0462 (14)	0.0009 (12)	−0.0024 (13)	0.0008 (13)
C10	0.0387 (15)	0.0545 (17)	0.0415 (13)	−0.0029 (12)	−0.0001 (13)	−0.0006 (13)
C11	0.064 (2)	0.0643 (18)	0.0679 (19)	0.0148 (17)	0.0105 (16)	−0.0003 (16)
C12	0.069 (2)	0.0561 (18)	0.076 (2)	0.0125 (16)	0.0017 (18)	0.0033 (15)
C13	0.059 (2)	0.0462 (18)	0.084 (2)	−0.0008 (15)	−0.0015 (17)	0.0065 (16)
C14	0.0503 (18)	0.0530 (18)	0.0792 (19)	−0.0029 (14)	−0.0015 (17)	0.0058 (15)
C15	0.098 (3)	0.087 (3)	0.102 (3)	0.030 (2)	0.018 (2)	0.046 (2)
C16	0.207 (6)	0.156 (5)	0.150 (5)	0.099 (5)	0.068 (5)	0.061 (4)
C17	0.125 (4)	0.053 (2)	0.213 (5)	−0.012 (2)	−0.034 (3)	−0.014 (3)
C18	0.075 (2)	0.0528 (18)	0.0624 (19)	0.0052 (17)	−0.0019 (19)	0.0097 (16)
C19	0.132 (3)	0.054 (2)	0.123 (3)	−0.0241 (19)	−0.047 (3)	0.0172 (19)
C20	0.0569 (18)	0.0573 (17)	0.0462 (14)	−0.0039 (14)	−0.0069 (13)	−0.0065 (13)

### Geometric parameters (Å, °)

**Table:**

O1—C3	1.201 (3)	C9—C10	1.541 (3)
O2—C18	1.196 (3)	C9—H9	0.9800
O3—C18	1.354 (3)	C10—C20	1.534 (3)
O3—C6	1.457 (3)	C11—C12	1.523 (3)
O4—C7	1.429 (3)	C11—H11A	0.9700
O4—C8	1.461 (3)	C11—H11B	0.9700
C1—C10	1.519 (3)	C12—C13	1.532 (4)
C1—C2	1.534 (4)	C12—H12A	0.9700
C1—H1A	0.9700	C12—H12B	0.9700
C1—H1B	0.9700	C13—C15	1.490 (4)
C2—C3	1.474 (4)	C13—C17	1.533 (4)
C2—H2A	0.9700	C13—C14	1.546 (4)
C2—H2B	0.9700	C14—H14A	0.9700
C3—C4	1.526 (4)	C14—H14B	0.9700
C4—C18	1.514 (4)	C15—C16	1.263 (6)
C4—C5	1.523 (3)	C15—H15	0.9300
C4—C19	1.534 (4)	C16—H16A	0.91 (2)
C5—C6	1.516 (4)	C16—H16B	0.97 (2)
C5—C10	1.536 (3)	C17—H17A	0.9600
C5—H5	0.9800	C17—H17B	0.9600
C6—C7	1.499 (3)	C17—H17C	0.9600
C6—H6	0.9800	C19—H19A	0.9600
C7—C8	1.458 (3)	C19—H19B	0.9600
C7—H7	0.9800	C19—H19C	0.9600
C8—C14	1.498 (3)	C20—H20A	0.9600
C8—C9	1.521 (3)	C20—H20B	0.9600
C9—C11	1.533 (3)	C20—H20C	0.9600
			
C18—O3—C6	110.1 (2)	C20—C10—C5	113.6 (2)
C7—O4—C8	60.57 (15)	C1—C10—C9	111.4 (2)
C10—C1—C2	111.3 (2)	C20—C10—C9	110.3 (2)
C10—C1—H1A	109.4	C5—C10—C9	105.44 (18)
C2—C1—H1A	109.4	C12—C11—C9	108.9 (2)
C10—C1—H1B	109.4	C12—C11—H11A	109.9
C2—C1—H1B	109.4	C9—C11—H11A	109.9
H1A—C1—H1B	108.0	C12—C11—H11B	109.9
C3—C2—C1	118.0 (3)	C9—C11—H11B	109.9
C3—C2—H2A	107.8	H11A—C11—H11B	108.3
C1—C2—H2A	107.8	C11—C12—C13	113.0 (2)
C3—C2—H2B	107.8	C11—C12—H12A	109.0
C1—C2—H2B	107.8	C13—C12—H12A	109.0
H2A—C2—H2B	107.1	C11—C12—H12B	109.0
O1—C3—C2	119.6 (3)	C13—C12—H12B	109.0
O1—C3—C4	119.7 (3)	H12A—C12—H12B	107.8
C2—C3—C4	120.6 (3)	C15—C13—C12	108.3 (3)
C18—C4—C5	102.3 (2)	C15—C13—C17	112.9 (3)
C18—C4—C3	111.8 (2)	C12—C13—C17	109.0 (3)
C5—C4—C3	114.9 (2)	C15—C13—C14	108.6 (2)
C18—C4—C19	107.7 (3)	C12—C13—C14	109.8 (2)
C5—C4—C19	112.9 (2)	C17—C13—C14	108.2 (3)
C3—C4—C19	107.1 (3)	C8—C14—C13	114.2 (2)
C6—C5—C4	102.9 (2)	C8—C14—H14A	108.7
C6—C5—C10	113.2 (2)	C13—C14—H14A	108.7
C4—C5—C10	114.10 (19)	C8—C14—H14B	108.7
C6—C5—H5	108.8	C13—C14—H14B	108.7
C4—C5—H5	108.8	H14A—C14—H14B	107.6
C10—C5—H5	108.8	C16—C15—C13	129.5 (5)
O3—C6—C7	108.9 (2)	C16—C15—H15	115.2
O3—C6—C5	104.77 (18)	C13—C15—H15	115.2
C7—C6—C5	116.4 (2)	C15—C16—H16A	118 (2)
O3—C6—H6	108.9	C15—C16—H16B	111 (2)
C7—C6—H6	108.9	H16A—C16—H16B	131 (3)
C5—C6—H6	108.9	C13—C17—H17A	109.5
O4—C7—C8	60.81 (15)	C13—C17—H17B	109.5
O4—C7—C6	113.7 (2)	H17A—C17—H17B	109.5
C8—C7—C6	120.3 (2)	C13—C17—H17C	109.5
O4—C7—H7	116.7	H17A—C17—H17C	109.5
C8—C7—H7	116.7	H17B—C17—H17C	109.5
C6—C7—H7	116.7	O2—C18—O3	120.1 (3)
C7—C8—O4	58.63 (14)	O2—C18—C4	129.7 (3)
C7—C8—C14	119.9 (2)	O3—C18—C4	110.2 (3)
O4—C8—C14	114.8 (2)	C4—C19—H19A	109.5
C7—C8—C9	118.7 (2)	C4—C19—H19B	109.5
O4—C8—C9	115.74 (19)	H19A—C19—H19B	109.5
C14—C8—C9	116.1 (2)	C4—C19—H19C	109.5
C8—C9—C11	110.2 (2)	H19A—C19—H19C	109.5
C8—C9—C10	109.59 (19)	H19B—C19—H19C	109.5
C11—C9—C10	116.4 (2)	C10—C20—H20A	109.5
C8—C9—H9	106.7	C10—C20—H20B	109.5
C11—C9—H9	106.7	H20A—C20—H20B	109.5
C10—C9—H9	106.7	C10—C20—H20C	109.5
C1—C10—C20	109.82 (19)	H20A—C20—H20C	109.5
C1—C10—C5	106.3 (2)	H20B—C20—H20C	109.5
			
C10—C1—C2—C3	−30.0 (4)	C2—C1—C10—C20	−60.7 (3)
C1—C2—C3—O1	174.5 (3)	C2—C1—C10—C5	62.5 (3)
C1—C2—C3—C4	−6.6 (4)	C2—C1—C10—C9	176.9 (2)
O1—C3—C4—C18	−57.0 (4)	C6—C5—C10—C1	−179.6 (2)
C2—C3—C4—C18	124.1 (3)	C4—C5—C10—C1	−62.4 (3)
O1—C3—C4—C5	−173.1 (3)	C6—C5—C10—C20	−58.8 (3)
C2—C3—C4—C5	8.0 (4)	C4—C5—C10—C20	58.5 (3)
O1—C3—C4—C19	60.7 (4)	C6—C5—C10—C9	62.1 (3)
C2—C3—C4—C19	−118.2 (3)	C4—C5—C10—C9	179.3 (2)
C18—C4—C5—C6	28.8 (2)	C8—C9—C10—C1	−179.5 (2)
C3—C4—C5—C6	150.2 (2)	C11—C9—C10—C1	54.6 (3)
C19—C4—C5—C6	−86.7 (3)	C8—C9—C10—C20	58.3 (2)
C18—C4—C5—C10	−94.2 (3)	C11—C9—C10—C20	−67.5 (3)
C3—C4—C5—C10	27.2 (3)	C8—C9—C10—C5	−64.7 (2)
C19—C4—C5—C10	150.3 (3)	C11—C9—C10—C5	169.5 (2)
C18—O3—C6—C7	145.2 (2)	C8—C9—C11—C12	47.6 (3)
C18—O3—C6—C5	20.1 (3)	C10—C9—C11—C12	173.2 (2)
C4—C5—C6—O3	−30.2 (2)	C9—C11—C12—C13	−67.5 (3)
C10—C5—C6—O3	93.4 (2)	C11—C12—C13—C15	142.6 (3)
C4—C5—C6—C7	−150.5 (2)	C11—C12—C13—C17	−94.1 (3)
C10—C5—C6—C7	−26.9 (3)	C11—C12—C13—C14	24.2 (3)
C8—O4—C7—C6	112.7 (2)	C7—C8—C14—C13	153.1 (2)
O3—C6—C7—O4	166.57 (18)	O4—C8—C14—C13	86.4 (3)
C5—C6—C7—O4	−75.4 (3)	C9—C8—C14—C13	−52.9 (3)
O3—C6—C7—C8	−124.6 (2)	C15—C13—C14—C8	−84.6 (3)
C5—C6—C7—C8	−6.5 (3)	C12—C13—C14—C8	33.6 (3)
C6—C7—C8—O4	−101.9 (3)	C17—C13—C14—C8	152.5 (3)
O4—C7—C8—C14	−102.5 (3)	C12—C13—C15—C16	115.8 (5)
C6—C7—C8—C14	155.6 (2)	C17—C13—C15—C16	−5.0 (6)
O4—C7—C8—C9	104.2 (2)	C14—C13—C15—C16	−125.0 (5)
C6—C7—C8—C9	2.3 (3)	C6—O3—C18—O2	176.6 (3)
C7—O4—C8—C14	111.2 (2)	C6—O3—C18—C4	−1.0 (3)
C7—O4—C8—C9	−109.3 (2)	C5—C4—C18—O2	164.5 (3)
C7—C8—C9—C11	163.7 (2)	C3—C4—C18—O2	41.0 (4)
O4—C8—C9—C11	−129.6 (2)	C19—C4—C18—O2	−76.3 (4)
C14—C8—C9—C11	9.4 (3)	C5—C4—C18—O3	−18.1 (3)
C7—C8—C9—C10	34.3 (3)	C3—C4—C18—O3	−141.6 (2)
O4—C8—C9—C10	101.1 (2)	C19—C4—C18—O3	101.0 (3)
C14—C8—C9—C10	−119.9 (2)		

### Hydrogen-bond geometry (Å, °)

**Table:**

*D*—H···*A*	*D*—H	H···*A*	*D*···*A*	*D*—H···*A*
C2—H2B···O2^i^	0.97	2.58	3.356 (5)	137
C7—H7···O4^ii^	0.98	2.58	3.164 (4)	118
C11—H11A···O1^iii^	0.97	2.55	3.420 (5)	149
C14—H14B···O1^iv^	0.97	2.52	3.486 (5)	178

Symmetry codes: (i) *x*−1, *y*, *z*; (ii) *x*+1/2, −*y*+3/2, −*z*+1; (iii) −*x*, *y*+1/2, −*z*+3/2; (iv) −*x*+1, *y*+1/2, −*z*+3/2.
